# Long-term effect of temperature on honey yield and honeybee phenology

**DOI:** 10.1007/s00484-016-1293-x

**Published:** 2016-12-24

**Authors:** Aleksandra Langowska, Michał Zawilak, Tim H. Sparks, Adam Glazaczow, Peter W. Tomkins, Piotr Tryjanowski

**Affiliations:** 1 Institute of Zoology, Poznań University of Life Sciences, Wojska Polskiego 71 C, 60-625 Poznań, Poland; 2 Dziadoszańska 42, 54-152, Wrocław, Poland; 3 sigma & Faculty of Engineering, Environment and Computing, Coventry University, Priory Street, Coventry, CV1 5FB UK; 4 Department of Systematic Zoology, Institute of Environmental Biology, Adam Mickiewicz University, Umultowska 89, 61-614 Poznań, Poland; 5 11 Cherry Orchard, Hemel Hempstead, Hertfordshire, HP1 3NQ UK

**Keywords:** Honeybee phenology, Climate change, Honey production

## Abstract

There is growing concern about declines in pollinator species, and more recently reservations have been expressed about mismatch in plant-pollinator synchrony as a consequence of phenological change caused by rising temperatures. Long-term changes in honeybee *Apis mellifera* phenology may have major consequences for agriculture, especially the pollinator market, as well as for honey production. To date, these aspects have received only modest attention. In the current study, we examine honeybee and beekeeping activity in southern Poland for the period 1965–2010, supplemented by hive yields from a beekeeper in southern UK in the same period. We show that despite negative reports on honeybee condition, and documented climate change, the studied apiary managed to show a marked increase in honey production over the 46 year study period, as did that from the UK. The proportion of the annual yield originating from the first harvest decreased during the study period and was associated with rising temperatures in summer. Honeybee spring phenology showed strong negative relationships with temperature but no overall change through time because temperatures of key early spring months had not increased significantly. In contrast, increasing yields and an increased number of harvests (and hence a later final harvest and longer season) were detected and were related to rising temperatures in late spring and in summer.

## Introduction

Recent interest in honeybees has been focussed on mass losses of colonies and concerns about the pollination services that bees provide (Potts et al. 2010; Cressey et al. 2014; Polce et al. 2014). However, despite the fact that bee pollination services might be responsible for as much as the third of human nutrition, only a small proportion of bees are kept specifically with pollination in mind, and the pollinator crisis, until now, does not seem to have played a major part in the increase in the global stock of the honeybee (Aizen and Harder 2009; Breeze et al. 2014). In Europe and worldwide, honeybees are maintained mainly for honey production (Morse and Calderone 2000; Aizen and Harder 2009). Honey is an important component of the world’s economy and trade of natural honey was worth US$ 3.3 billion in 2011 (Gallai et al. 2009; FAO 2015). Over the last half century, there has been a steady increase in the global production of honey. The average productivity of each hive has also increased. The growth of production has outstripped the increase in bee colonies by more than a factor of two (Aizen and Harder 2009). Furthermore, the value of pollination by the honeybee may be worth from 30 to 100 times the value of honey and beeswax combined (FAO 2015). Globally, the value of insect pollination may exceed $300bn (Gallai et al. 2009; Lautenbach 2012) with the honeybee perhaps responsible for 80% of crop pollination overall (Prescott-Allen and Prescott-Allen 1990) and 100% in some intensive orchard crops.

Climate change appears to be a major concern for agriculture in general and may also have worrying implications for beekeeping. In particular, it has been listed as a possible contributor to the decline in pollinators, including honeybees, and a loss of synchronization between pollinator activity and flowering (Le Conte and Navajas 2008; Hegland et al. 2009; Lever et al. 2014). The warming aspect of climate change may have a substantial impact on the production of honey. Surprisingly, few data are available on the relationship between climate change and the biology of the honeybee (Scheifinger et al. 2005; Gordo and Sanz 2006; Sparks et al. 2010; Henneken et al. 2012). Data from apiaries collected over a long period are valuable in that they allow trends to be revealed and correlated with changes in weather, with extrapolation of results into the future. This in turn should help identify relevant measures needed to protect beekeepers against the impact of climate change. In this paper, we analyse how temperature influences various parameters concerning the production of honey. We focus on a record that has been meticulously collected over a period of 46 years in southern Poland. We supplement these data with an independent record of honey production from the southern UK for the same period.

## Materials and methods

Data were collected for the 46 years 1965–2010 in the vicinity of Wrocław, Poland (51°06′N 17°01′E), by the same person (MZ). The dominant form of land use around the apiary was mainly arable, but the area also comprised woodland patches, and especially linear woody features growing alongside drainage ditches and roads, and semi-natural vegetation growing in non-cropped habitats; for details see Orłowski and Nowak (2005).

The following phenological dates were collected: first cleansing flight, first inspection of hives, first honey harvest, final harvest, and harvest interval (last-first harvest date). For brevity, these are referred to throughout the text as phenology although we acknowledge that some are subject to human decision and are thus “false” phases sensu Schnelle (1955). First inspection of hives was performed as early as possible, i.e., when temperature reached about 15 °C. Similarly, first honey harvest was taken as soon as the honey combs were capped by bees, not waiting until honey supers were filled out totally, due to the risk that spring honey might crystallize (granulate) inside the comb before extraction. Last harvest took place when honey accumulation was slowing towards the end of the season, however again, it was performed as early as possible, in order not to delay colonies’ preparation for winter. The dates of intermediate harvests were not treated as phenological dates since timing of these is much more flexible. Honey yields, at each of a variable number of harvests per year, were recorded. This information was used to generate for each year the mean annual honey yield per hive, the mean first harvest honey yield per hive, and the proportion of the annual yield taken at the first harvest. Phenology data were recorded for 39 to 46 years; yield data were missing for 1965 (*n* = 45). An additional, independent, data series of mean annual honey yield per hive from a similar latitude (Harpenden, UK; 51°49′N 0°21′W) recorded by one beekeeper (PWT) was abstracted for 40 years in the common period (1965–2010). The UK area was also an arable landscape, with hives located on non-cropped habitat (e.g. scrub, field or wood margins). The hives at the two locations could be moved around, thus the areal extent served by the bees could vary. The main cropping change at both locations over the study period was the introduction of, and increase in, oilseed rape from the late 1970s, but more detailed data on land-use were not recorded. Bees were maintained in standard wooden and plywood hive boxes, i.e., modified Wielkopolski type in Poland and Smith type in the UK; however, at the Polish site different type of hives, including horizontal ones, were used for the first 5 years. At the Polish site in 1970, the local subspecies of bees was exchanged for Carniolan (*A. m. carnica*) bees, when the subspecies became available from government breeding stations. Since then regular re-queening was performed as the main swarming prevention practice.

Mean monthly temperatures (°C) and monthly precipitation sums (mm) were obtained from the E-OBS (Haylock et al. 2008) v12.0 0.25° gridded data set averaged for 50.75–51.25°N, 16.75–17.25°E and accessed via the KNMI Climate Explorer website (http://climexp.knmi.nl). Mean monthly temperatures (°C) and monthly precipitation sums (mm) for the Rothamsted (UK) met station (51°48′N 0°18′W) were obtained courtesy of Rothamsted Research.

Dates were converted to day of the year (1 = January 1, etc.) prior to analysis. Trends through time were estimated by regression of the variable on year. Temperature and precipitation responses were examined initially by Pearson correlations with preceding calendar monthly temperatures and precipitation and then by regression of variables on combinations of calendar monthly temperatures. Initial screening suggested that monthly precipitation was not a very important predictor compared to temperature; significant for just three variables, and only one survived as significant in models with temperature (see Results). The appropriate combination of months was taken as adjacent months that were significant in the initial screening based on correlation. A non-temperature trend in time (which may be related to changes in management etc.) may be confounded with a trend in temperature. Following the approach of Estrella et al. (2007), all regressions were repeated including an initial year term. The initial regression may overestimate the effect of temperature since it may also include (be confounded with), for example, management changes. The second regression tends to underestimate the effect of temperature since it only estimates fluctuations about a trend in temperature. The true effect lies in between the two estimates. Annual yield was related to phenology and temperature using stepwise multiple regression. The threshold for significance was taken as *P* = 0.05.

## Results

Trends in mean monthly temperatures and monthly precipitation sums for Wrocław and Rothamsted are summarized in Table 1. In Wrocław, there were significant increases in temperatures for each month from April to August, in addition mean annual temperature rose significantly by 0.0355 °C/year or an equivalent of 1.6 °C over the study period. In Rothamsted, significant increases in temperature occurred for each month from March to September and in November. There was only one significant increase in precipitation (Wrocław in March), although that for Rothamsted in August also came close to being significant (*P* = 0.051).

**Table 1:** Trends in mean monthly temperature (°C) and monthly precipitation sums (mm) recorded at Wrocław, Poland and Rothamsted, UK 1958–2010. Mean values are shown together with regression coefficients and standard errors (*b* ± SE) from regressions of the variables on year and an indication of statistical significance (*P*). Significant results (*P* < 0.05) are shown in italics

	Wrocław						Rothamsted					
	Temperature			Precipitation			Temperature			Precipitation		
	Mean	*b* ± SE	*P*	Mean	*b* ± SE	*P*	Mean	*b* ± SE	*P*	Mean	*b* ± SE	*P*
Jan	−1.0	0.038 ± 0.036	0.301	26	−0.01 ± 0.16	0.970	3.7	0.028 ± 0.019	0.158	68	0.19 ± 0.34	0.582
Feb	0.2	0.035 ± 0.033	0.299	25	0.05 ± 0.14	0.728	3.8	0.040 ± 0.021	0.060	52	0.13 ± 0.33	0.687
Mar	3.7	0.020 ± 0.023	0.374	*29*	*0.40 ± 0.17*	*0.024*	*5.9*	*0.042 ± 0.014*	*0.005*	52	−0.13 ± 0.30	0.670
Apr	*8.4*	*0.057 ± 0.013*	*<0.001*	35	−0.24 ± 0.18	0.199	*8.0*	*0.048 ± 0.010*	*<0.001*	54	0.04 ± 0.34	0.897
May	*13.5*	*0.044 ± 0.015*	*0.005*	59	−0.29 ± 0.34	0.400	*11.3*	*0.033 ± 0.012*	*0.007*	55	−0.05 ± 0.34	0.880
Jun	*16.6*	*0.033 ± 0.013*	*0.014*	70	−0.53 ± 0.38	0.167	*14.3*	*0.032 ± 0.013*	*0.018*	54	−0.34 ± 0.36	0.347
Jul	*18.3*	*0.063 ± 0.016*	*<0.001*	88	−0.05 ± 0.55	0.924	*16.4*	*0.044 ± 0.014*	*0.004*	49	0.06 ± 0.27	0.827
Aug	*17.9*	*0.057 ± 0.012*	*<0.001*	68	−0.31 ± 0.49	0.528	*16.4*	*0.039 ± 0.013*	*0.005*	61	0.70 ± 0.35	0.051
Sep	13.7	0.018 ± 0.015	0.249	46	−0.02 ± 0.31	0.953	*13.9*	*0.031 ± 0.011*	*0.009*	58	−0.25 ± 0.42	0.551
Oct	9.0	0.011 ± 0.019	0.560	35	−0.32 ± 0.28	0.270	10.5	0.014 ± 0.016	0.387	74	0.67 ± 0.47	0.163
Nov	4.0	0.027 ± 0.020	0.188	38	−0.22 ± 0.18	0.239	*6.4*	*0.043 ± 0.013*	*0.003*	73	0.61 ± 0.38	0.120
Dec	0.3	0.002 ± 0.027	0.952	32	−0.08 ± 0.21	0.690	4.3	−0.001 ± 0.018	0.944	70	−0.04 ± 0.38	0.915

**Fig. 1:**
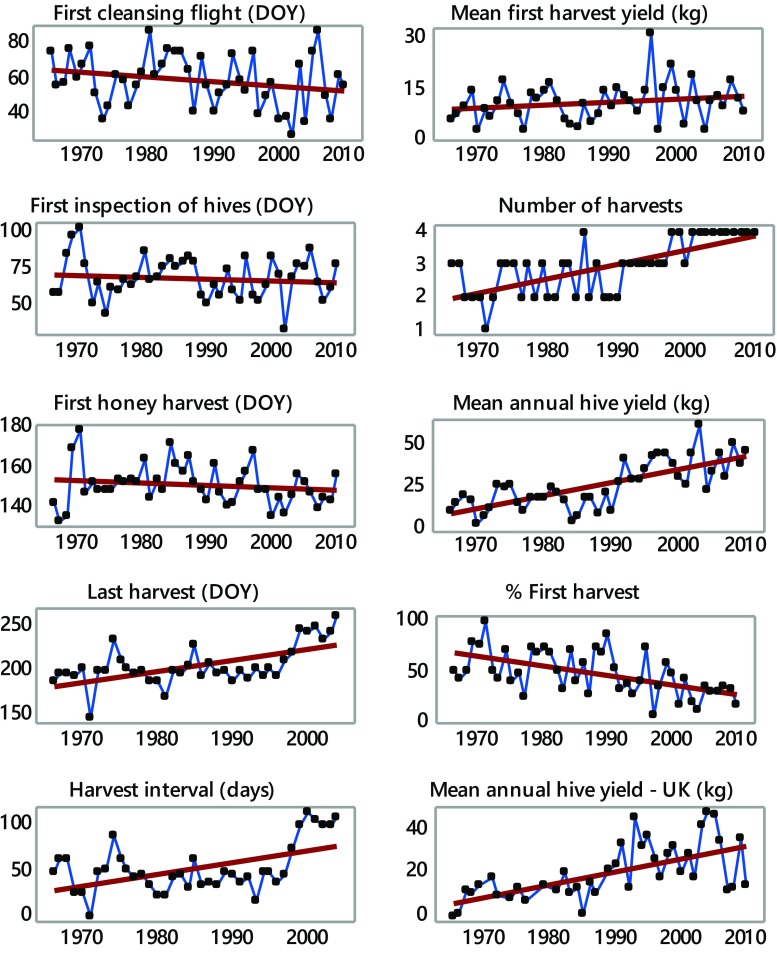
Trends in phenology (*DOY* Day of Year) and in yield variables. Regression lines superimposed

Trends in phenology and yield are summarized in Table 2 and series are plotted in Fig. 1. There were no significant changes in spring phenology, but the final harvest date had become significantly later, and hence the harvest interval (last-first harvest dates) also significantly increased, both by 1.2 days/year (equivalent to 55 days over the study period). The number of harvests increased significantly, annual yield increased by an average of 0.76 kg/year (*P* < 0.001), but there was no significant change in first harvest yield. Consequently, the contribution of the first harvest to annual yield declined by 0.9%/year (*P* < 0.001). Because of a possible outlier (1996 first harvest yield, identified in comparison to prevailing temperature), analyses were rerun excluding this outlier, but with only modest differences in results drawing no change to conclusions. The annual yield per hive also increased significantly at the UK site by 0.58 kg/year (*P* < 0.001).

**Table 2:** Trends in honeybee phenology and yield in Wrocław, Poland, 1965–2010. Per annum changes in the variables (*b*) are displayed with SE. *n* indicates numbers of years of data

	*n*	Mean	*b* ± SE	*R* ^2^(%)	*P*
First cleansing flight	46	58 (Feb 27)	−0.25 ± 0.16	5.4	0.119
First inspection of hives	45	68 (Mar 9)	−0.11 ± 0.16	1.1	0.486
First harvest	45	151 (May 31)	−0.12 ± 0.11	6.6	0.297
Last harvest	39	206 (July 25)	1.21 ± 0.27	35.0	<0.001
Interval from first to last harvest (days)	39	51	1.22 ± 0.34	24.4	0.001
Number of harvests^a^	45	3.0	0.015 ± 0.007	48.8	0.023
Mean hive annual yield (kg)	45	25.7	0.76 ± 0.10	55.1	<0.001
Mean hive annual yield UK	40	19.8	0.58 ± 0.12	40.0	<0.001
Mean hive first harvest yield (kg)	45	10.8	0.10 ± 0.06	5.0	0.138
% of annual yield taken at first harvest	45	47.8	−0.91 ± 0.22	29.1	<0.001

^a^Poisson regression, deviance *R* ^2^ reported

**Fig. 2:**
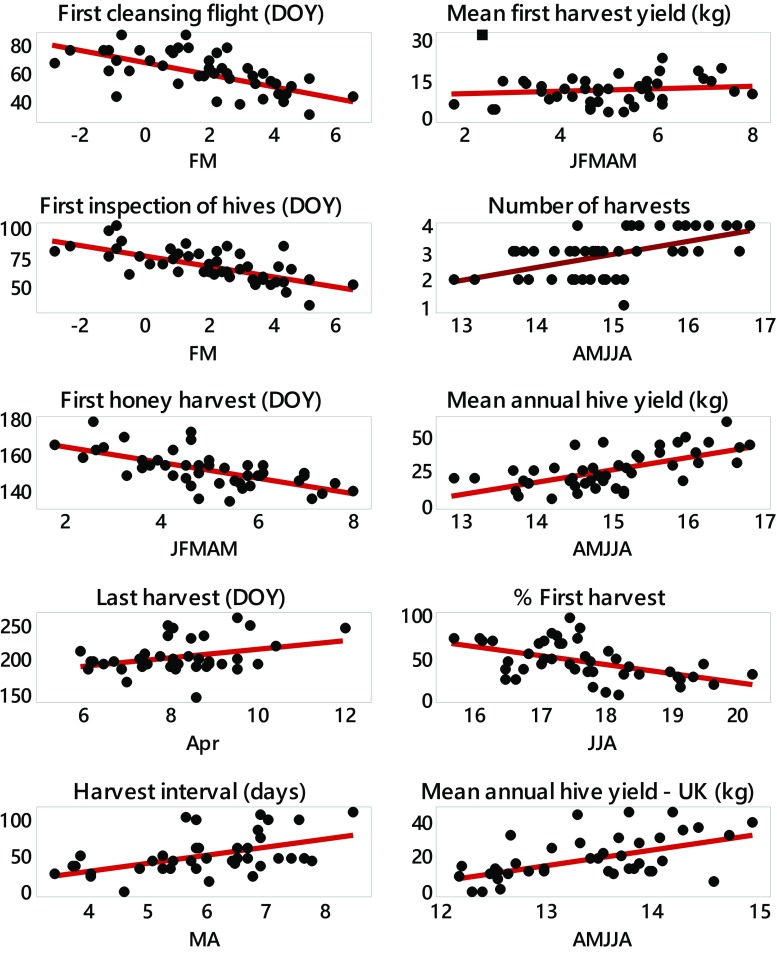
Relationships with temperature for phenology (*DOY* Day of Year) and yield variables. All *x*-axis units are °C and are means of calendar months using the following abbreviations (*J* January, *F* February, *M* March, *A/Apr* April, *M* May, *J* June, *J* July, *A* August). A possible outlier in first harvest yield is indicated by a *square* (see text for details). Regression lines superimposed

Regressions on temperatures revealed significant relationships for all phenological variables (Table 3, Fig. 2). Temperature responses for the spring variables ranged from −4.3 to −4.6 days/°C. Annual yield was significantly and positively related to mid-year temperatures (April–August); a 1 °C increase associated with an 8.97 kg increase in yield. A remarkably similar response (8.71 kg/°C) to temperatures of the same months was found for the UK; the only variable for which a significant, and in this case negative, effect of precipitation was found. No significant model was found for first harvest yield. However, closer inspection suggested that this may have been influenced by one outlier (i.e., 1996; Fig. 2). Reanalysis without this point suggested a significant regression on January–May mean temperature of 1.18 ± 0.49 kg/°C (*R* ^2^ = 12.3%, *P* = 0.020). Curiously, the % contribution of first harvest yield was significantly related to June–August temperature, but this is caused by warmer weather in those months boosting subsequent harvests, thus reducing the contribution of the first harvest.

**Table 3:** Relationships of honeybee phenology and yield with temperature and precipitation in Wrocław, Poland 1965–2010. Changes in the variables per 1 °C/mm (*b*) are displayed with SE. For sample size, see Table 2. Models were repeated having initially fitted a year term (*incl. year*) as a conservative estimate of temperature effect (see text for details). The column headed months indicates which months’ temperatures were averaged for use in regression (*J* January, *F* February, *M* March, *A*/*Apr* April, *M* May, *J* June, *J* July, *A* August). *P*5 May precipitation

	Months	*b* ± SE	*R* ^2^(%)	*P*
First cleansing flight	FM incl. year	−4.26 ± 0.79 –4.11 ± 0.80	39.7 41.3	<0.001 <0.001
First inspection of hives	FM incl. year	−4.57 ± 0.73 –4.55 ± 0.74	47.7 47.8	<0.001 <0.001
First harvest	JFMAM incl. year	−4.48 ± 0.77 –4.61 ± 0.82	44.0 44.3	<0.001 <0.001
Last harvest	Apr incl. year	6.20 ± 2.75 2.40 ± 2.58	12.0 36.5	0.030 <0.001
Interval from first to last harvest (days)	MA incl. year	11.14 ± 3.10 8.53 ± 2.99	25.9 39.2	0.001 <0.001
Number of harvests [Poisson]^a^	AMJJA incl. year	0.17 ± 0.09 0.04 ± 0.13	30.0 49.5	0.074 0.072
Mean hive annual yield (kg)	AMJJA incl. year	8.97 ± 1.68 3.35 ± 1.94	39.8 58.0	<0.001 <0.001
Mean hive annual yield UK (kg)	AMJJA P5 incl. year	8.71 ± 2.14 –0.11 ± 0.05 1.08 ± 2.55	38.0 38.0 52.0	<0.001^b^ 0.041^b^ <0.001
Mean hive first harvest yield (kg)	JFMAM incl. year	0.51 ± 0.59 0.26 ± 0.62	1.7 5.4	0.388 0.675
% of annual yield taken at first harvest	JJA incl. year	−10.82 ± 2.78 –6.02 ± 3.41	26.0 34.0	<0.001 <0.001

^a^Poisson regression, deviance *R* ^2^ reported

^b^Significance of each term, overall model *P* < 0.001

The second run of regressions, including an initial year term, barely affected the temperature response for first cleaning flight, first inspection or first harvest date, despite the latter two arguably being “false” phases (Table 3). The influence of temperature on last harvest date was modified to be between 2 days/°C (regression with a year term) and 6 days/°C (regression without a year term). Likewise, the true effect of temperature on the harvest interval might be reduced. The influence of temperature on yield could be seriously reduced, indicating some unspecified management changes enhancing yields over the 46 years of study. Removing 1996 from the analysis of mean first harvest yield after initially fitting year still resulted in a significant temperature effect (*b* = 1.07 ± 0.53, *R* ^2^ = 12.3%, *P* = 0.020). Temperature remained influential on the percentage of yield taken at the first harvest, although a reduced coefficient was apparent after fitting an initial year term.

Annual yield was examined against potential predictors of first cleansing flight, first inspection of hives, first harvest date, last harvest date, year, and temperatures for each of the 8 months January to August. A highly significant model (*R* ^2^ = 63.2%, *P* < 0.001) was obtained with three variables: year (*b* = 0.61 ± 0.12, *P* < 0.001), June mean temperature (*b* = 3.71 ± 1.13 *P* = 0.002), and first inspection of hives (*b* = −0.21 ± 0.09, *P* = 0.028). These coefficients suggest an improvement over time of approximately 0.6 kg/year, that a 1 **°**C increase in June temperature was associated with a 3.7 kg increase in yield, and that earlier first inspection of hives by about 5 days was associated with a 1 kg increase in mean annual yield.

## Discussion

Numerous studies have shown an advance in the timing of flowering of many plants driven by climate change (e.g. see Menzel et al. 2006 for a continent-scale summary), including in Poland (Sparks et al. 2011). The possible mismatch between phenology of plants and honeybees, and the introduction of new cultivars of annual crops bred in response to a changing climate are both of concern to beekeepers (Kołtowski 2002; Gordo and Sanz 2006; Lever et al. 2014). It has been documented that recently honeybee colonies were not able to develop early in the year, and the mass spring emergence of worker bees appeared half way or later into the winter oilseed rape flowering period (Kołtowski 2002). This stimulates bees to swarm (founding new colonies by division) that seriously reduces the honey harvest. Therefore, early activity may be crucial in order for honeybees to take advantage of spring nectar flows. The first phenological sign of spring activity in a honeybee colony is the first cleansing flight that bees perform to void after the winter months. Our research confirms this date is dependent on late winter temperature (February to March). The first hive inspection is similarly influenced, presumably because the inspector is aware of the warmer and earlier spring and the need for an earlier inspection under such conditions. Interestingly, while mid-year temperatures increased in the study period, there was no significant change in these early months (Table 1). Consequently, it is not surprising that there was no significant change in the date of first hive inspection. However, our findings, in accordance with other research (Scheifinger et al. 2005; Gordo and Sanz 2006; Sparks et al. 2010), suggest an advance of honeybee activity, but against a background of great variability in timing such that no significant change could be detected.

The honey yield from the first harvest did not change significantly during the study period, suggesting that yield is taken off when it is economic to do so rather than being fixed to a calendar date. There had been no advance in the date of first harvest. However, there was evidence that both first harvest date could be advanced by, and the first harvest yield boosted by, increased temperature from January to May. Significant warming has only been experienced towards the end of this 5 month period. Since the overall yield has increased, the proportion of annual yield coming from the first harvest has thus decreased. The higher temperatures we observed in spring (e.g., April, May) could shorten the flowering period of individual plants and advance the flowering date of both early and late spring plant species (Sparks et al. 2011). Therefore, the flowering of the main spring nectar source plants may totally (fruit trees, oilseed rape, dandelion *Taraxacum* spp., some willow species *Salix* spp.) or partially (oilseed rape and locust tree *Robinia pseudoacacia*) overlap. This can result in an overflow of nectar and bee colonies may not be able to exploit all of the available resources. Foraging resources are limited in number, particularly in spring when the colony builds up strength. Therefore, bees respond to an overflow of nectar by multiplying and preparing to swarm, which limits honey production. To tackle this, the beekeeper would have to keep more hives to increase overall production. Such a situation may also handicap the production of varietal (monofloral) honey, which is of a higher economic value for the beekeeper (Krell 1996).

In contrast, annual yield did increase over time and nearly trebled during the study period. These results were confirmed from an independent record from the UK. Stepwise regression analysis suggested that, in addition to improvements over time, increases in temperature in June, and an early season (as denoted by earlier first inspection of hives) would increase annual yield. Our results indicate that warmer temperatures later in the season boost numbers and yields of later harvests thus resulting in a reduction in the % contribution of the first harvest to annual yield. Although the influence of climate change on pollinators has recently become a global concern, the studied system appears to be surprisingly successful with no obvious adverse effects detected to date. In fact, there has been a huge increase in yield at both Polish and UK sites. Bees can take advantage of the fact that spring nectar and pollen flow stimulate colony growth, therefore are better prepared to explore summer flows. Higher temperatures also extend the beekeeping season, therefore, bees may explore new flows. These include invasive plant species, as was the case of the studied apiary that managed to produce honey from the nectar of goldenrod *Solidago canadensis*, a plant very common in southern Poland (Dajdok and Wuczyński 2008).

Precipitation effects in our study did not seem to be important and temperature effects dominated. This is consistent with what we know about flower phenology in non water-limited environments. This does not mean that precipitation does not influence beekeeping but rather that the scale (monthly sums) of data was too crude to realistically be expected to be influential. A soil water deficit could hasten the end of flower nectar production, and long periods of heavy precipitation could reduce bee activity or damage flowers. However, appropriate data at the required temporal resolution were not available to the authors to investigate further. Some of the changes shown in Fig. 1 may exhibit a step-like change in the vicinity of the late 1980s and, if so, would be compatible with the theory of a climate regime shift in this period (Reid et al. 2016); all the more, since there was no change in management practice at this time. Certainly, the abrupt changes are not apparent in the temperature plots in Fig. 2. Investigating this phenomenon with our bee data is beyond the scope of the current paper.

We might also expect, and have detected, improvements over time as a consequence of beekeeper experience and better quality of bees available (Le Conte and Navajas 2008). It is not possible to fully separate these effects from those of temperature in a period of increasing mid-year temperatures. As a consequence, we have generated estimates of temperature effects confounded with non-climate related trends, and after eliminating trends. The former may overestimate, and the latter underestimate the true influence of temperature. Given the trends over the last 46 years, we therefore believe that it should be possible, to a certain degree, for beekeepers to adapt to climate change by making appropriate management changes.
